# The effects of pulsed radiofrequency stimulation on mitochondrial function in neurons: a mini review

**DOI:** 10.3389/fpain.2026.1828597

**Published:** 2026-06-22

**Authors:** Lukas D. Linde, Paul Potgieter, Petra van Blerk, Corlius F. Birkill

**Affiliations:** Algiamed Technologies, Burnaby, BC, Canada

**Keywords:** bioenergetics, mitochondria, neuropathic pain, neuroregeneration, pulsed radiofrequency (PRF)

## Abstract

Pulsed radiofrequency (PRF) stimulation is a widely used clinical intervention for neuropathic pain. Unlike continuous radiofrequency (CRF) ablation, PRF is non-destructive, yet its precise mechanism of action remains under debate. Emerging evidence suggests a fundamental role of mitochondrial modulation in driving both analgesia and nerve regeneration. To synthesize the literature investigating PRF effects on neuronal mitochondrial function, a scoping review was conducted across five databases (MEDLINE, Embase, CENTRAL, Web of Science, PubMed) from inception to February 2, 2026. From 141 initial records, 10 experimental studies met the inclusion criteria for narrative synthesis. Four studies comparing PRF with CRF confirmed that while CRF induces severe mitochondrial pathology (swelling, cristolysis, and necrosis), PRF preserves mitochondrial ultrastructure. The remaining six studies identified specific bioenergetic mechanisms. PRF was found to actively decrease mitochondrial membrane potential (*ΔΨm*) while increasing cytosolic ATP, effectively reversing the bioenergetic profile associated with central sensitization. Furthermore, PRF treatment was associated with mitochondrial hyperplasia; while hypothesized to represent compensatory biogenesis supporting structural repair, confirmation of these exact pathways is still needed. PRF stimulation appears to exert its effects, in part, through multiple mitochondrial mechanisms: an acute analgesic phase mediated by depolarizing the mitochondrial membrane and reducing excitotoxic calcium influx, and a sub-acute neuroregenerative phase bioenergetically supported by the neuro-glial metabolic unit. Ultimately, further exploration into the specific mechanisms by which neuromodulation impacts mitochondrial health and function in neuropathies is warranted.

## 1 Introduction

Pulsed radiofrequency (PRF) stimulation is an interventional pain therapy originally devised as a neuromodulatory technique for the dorsal root ganglia (DRG) and trigeminal ganglia (1). Briefly, PRF is defined by the precise delivery of short, intermittent bursts of high frequency alternating current, typically around 500 kHz, via a specialized needle electrode placed adjacent to the target nerve. This creates a highly concentrated, fluctuating electrical field around the neural tissue without generating sustained frictional heat. Developed as a non-destructive alternative to continuous radiofrequency (CRF) ablation, PRF marked a significant advance in pain management. Historically, CRF employed thermal energy to intentionally lesion nerves to alleviate pain. In contrast, PRF applies an electromagnetic field to neural tissue in short bursts, allowing heat to dissipate between pulses. This maintains tissue temperature below 42 °C, avoiding the thermal coagulation and structural damage associated with continuous RF. While a robust body of evidence, including randomized controlled trials and reviews, supports the clinical efficacy of PRF for neuropathic pain (2–4), diabetic peripheral neuropathy (5–7), and other peripheral neuropathies (8), the precise cellular mechanisms underlying these analgesic effects remain poorly defined.

The prevailing theoretical framework for PRF's mechanism centers on anti-inflammatory effects and the modulation of oxidative stress (1, 9). It is hypothesized that inflammatory neuropathies are driven by cellular stress, where increased production of free radicals leads to oxidative stress, inflammation, and sympathetic hyperactivity. Current theories postulate that PRF electromagnetic fields may influence radical pair recombination, thereby interrupting the cycle of oxidative stress (1). Indeed, reduced levels of downstream inflammatory markers (e.g., TNF-ɑ, IL-6) have been observed following PRF treatment (9).

However, the “anti-inflammatory” hypothesis alone is insufficient. It fails to explain the biophysical transduction mechanism by which electromagnetic fields influence free radical dynamics or drive structural repair. Notably, recent reports describe ultrastructural changes in peripheral nerves following PRF stimulation that are indicative of neural regeneration (3). This raises the possibility that PRF exerts its effects through a central regulator of cellular energy and repair: the mitochondria. As the primary generators of free radicals via energy metabolism and key regulators of neural repair and regeneration (10), mitochondria represent the most plausible link between electromagnetic stimulation and cellular recovery. Understanding the direct effects of PRF on mitochondrial function is therefore critical to elucidating the biological basis of PRF therapy.

This scoping review aims to systematically explore and map the recent literature investigating the specific effects of pulsed radiofrequency stimulation on mitochondrial function within neurons and the nervous system. Our specific objectives are to: 1) describe the characteristics of PRF parameters employed; 2) ascertain the specific mitochondrial functions or parameters assessed; 3) identify the neuronal or nervous system cell types and models utilized; and 4) outline any reported direct or indirect effects of PRF on mitochondrial function.

## 2 Methods

### 2.1 Search strategy

The objective of this scoping review was to summarize and evaluate currently available literature on the effects of PRF stimulation on mitochondrial function within neurons and the nervous system. A protocol was not prospectively registered for this review. However, the review was conducted in accordance with the PRISMA-ScR (Preferred Reporting Items for Systematic Reviews and Meta-Analyses extension for Scoping Reviews) guidelines to ensure methodological rigor and transparency (11). Randomized controlled trials (RCTs), quasi-experimental studies, and observational studies were included in this search. To guide the systematic search of literature, the Problem, Intervention, Control, Outcome, Study Design (PICOS) strategy was used to address our primary question: Does pulsed radiofrequency stimulation affect mitochondrial function within neurons? A summary of our participants, intervention, comparisons, outcomes, and study types included in the review, in accordance with PICOS are outlined in Supplementary Table 1. Inclusion criteria encompassed experimental studies in animal and human models that examined the effects of pulsed radiofrequency stimulation on mitochondrial function within neurons or neural tissue. Included studies were published in English. Review articles, case studies, case reports, and clinical trial registries were excluded to focus on primarily experimental evidence.

The following databases were searched for relevant studies: MEDLINE (via Ovid 1946-2026); EMBASE (via Ovid 1974-2026); CENTRAL (via Ovid); Web of Science Core Collection (via Clarivate 1900-2026); PubMed (via National Library of Medicine 1946-2026). Included references were also hand-searched for relevant studies. The full search strategy for MEDLINE via Ovid is provided as an example: [exp Radiofrequency Therapy/ OR (pulsed radiofrequency OR radiofrequenc*).mp] AND [exp Mitochondria/ OR (mitochondria OR mitochondrial OR mitochondri*).mp] AND [exp nervous system/ OR (neuro* OR neuron* OR nerve*).mp]. Truncation symbols (*) were used to search for root variations. While no language restrictions were applied to the initial database search strategy, only articles published in English were included in the final review due to resource limitations regarding translation. Covidence systematic review software (Veritas Health Innovation, Melbourne, Australia. Available at http://www.covidence.org) was used to store, screen, and organize all the references acquired.

### 2.2 Screening and data extraction

Initial screening of titles and abstracts was conducted independently by two separate reviewers (LL and PP). In cases of disagreement, the article was re-evaluated by both reviewers to reach consensus. If consensus could not be achieved, a third reviewer (CFB) was consulted. Full-text screening was subsequently completed in the same fashion. Data extraction included four main domains: (1) article identification, (2) methodological characteristics (e.g., PRF stimulation parameters, animal vs. cell culture study, healthy vs. pain model), (3) main findings (e.g., effect of PRF on mitochondrial function), and (4) conclusions. Both LL and PP separately conducted all data extraction in Microsoft Excel. Data extraction tables were subsequently merged and examined for inconsistencies. Disagreements were resolved via consensus, through a third reviewer (CFB).

## 3 Results

**Figure 1:**
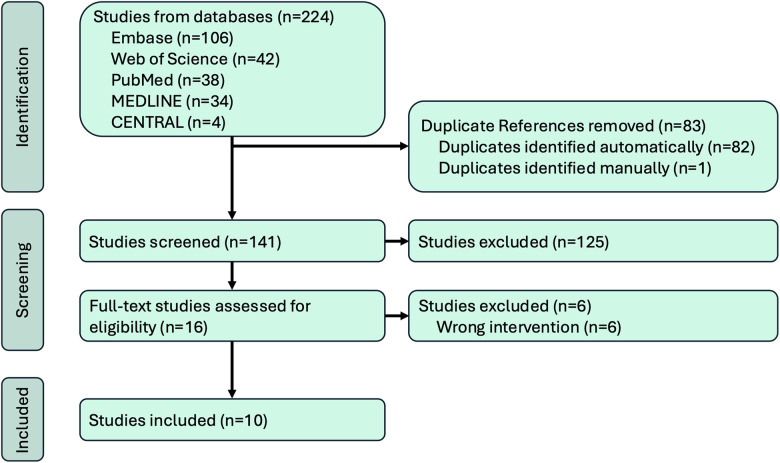
PRIMSA flow diagram.

Our final search of all databases, updated as of February 2, 2026, yielded 141 articles after removal of duplicates. Initial title and abstract screening yielded 16 full text articles sought for retrieval. Subsequent full-text screening yielded 10 articles for data extraction to be included in a narrative synthesis (Figure 1). Of these 10 studies, four studies compared CRF with PRF stimulation (12–15), while the remaining six examined PRF exclusively (16–21). Six studies used animal models [5 rat (12, 13, 15, 17, 21), 1 rabbit (16)], while the remaining four studies used *in vitro* tissue or cell culture models (14, 18–20). Details on PRF parameters used among included studies are summarized in Table 1.

**Table 1:** PRF stimulation parameters from ten (10) included studies.

Study	Model	RF parameters	Treatment duration	Key findings
Bayir et al. (12)	*In vivo* (rat); sciatic nerve incision & anastomosis.	40–60 V, 2 Hz, 20 ms pulse, 150–400 *Ω*, 42 °C.	120 s/day for up to 30 days.	Cellular: PRF better preserved the fine structure of mitochondria, neurotubules, and Schwann cell nuclei. Histological: PRF yielded significantly higher axon counts and larger diameters by day 30 (though control diameters were larger at day 15). Functional: No significant improvement in motor function (SFI) over 4 weeks compared to control.
Choi et al. (13)	*In vivo* (rat); healthy sciatic nerve (compared to sham/ control).	PRF: 42 °C, 240 pulses. CRF: 82 °C.	Single application. PRF: 120 s CRF: 60 s	Morphological/Cellular: PRF caused mild axonal damage, collagen swelling, and little mitochondrial swelling. CRF caused irreversible degeneration, myelinated axon collapse, absent cytoskeleton, severe mitochondrial and collagen damage. Inflammatory: PRF transiently upregulated pro-inflammatory markers (e.g., GFAP, TNF-α, IL-6), which recovered to normal by day 30. CRF caused persistent upregulation of these markers through day 30.
Erdine et al. (16)	*In vivo* (rabbit); healthy dorsal root ganglion (DRG) (compared to sham/ control).	PRF: 45 V, 2 Hz, 20 ms pulse. Target temperature <43 °C. CRF: 67 °C.	Single application. PRF: 120 s CRF: 60 s	Light Microscopy: No pathological differences or damage observed across any group. Electron Microscopy: PRF caused mild ultrastructural changes (enlarged ER cisterns, increased vacuoles) but preserved cell/nuclear membranes and mitochondria. CRF caused severe damage (giant fused vacuoles, degenerated mitochondria, and loss of nuclear membrane integrity). Myelinated and unmyelinated fibers remained structurally normal in both RF groups.
Erdine et al. (17)	*In vivo* (rat); healthy sciatic nerve (compared to contra-lateral sham).	PRF: 45 V, 1 ms pulse width, 2 Hz. Temp kept ≤42 °C.	Single application (exact duration not explicitly stated in text).	Cellular/Morphological: PRF caused significant ultrastructural damage to internal elements (mitochondria, microtubules, microfilaments), while outer axonal membranes showed no evident damage. Fiber Selectivity: Damage severity was size-dependent (unmyelinated C-fibers > A-delta fibers > A-beta fibers). This suggests PRF preferentially disrupts smaller pain-carrying pathways while relatively sparing tactile sensory fibers.
Laksono et al. (18)	*In vitro*; F11 cell line differentiated into DRG neurons. Sensitized via NMDA (80 μM).	500 kHz, 2 Hz, 20 ms pulse width. Needle positioned 500–1,000 μm from cells.	Single application. 360 s.	Sensitization: PRF significantly decreased NMDA-induced neuron sensitization, evidenced by lowered pERK expression. Mitochondrial/Metabolic: PRF restored metabolic homeostasis. It significantly decreased pathologically elevated mitochondrial membrane potential (*ΔΨ*m) and increased cytosolic ATP levels in sensitized neurons. Calcium: PRF-exposed neurons exhibited Ca2 + influx, but at significantly lower levels than unexposed sensitized neurons.
Laksono et al. (19)	*In vitro*; F11 cell line differentiated into DRG neurons. Healthy state.	Energy-matched comparison: 2 Hz: 20 ms pulse. 4 Hz: 10 ms pulse.	Single application. 360 s.	Safety/Homeostasis: When energy delivery was matched, 2 Hz better preserved the physiological properties of healthy neurons compared to 4 Hz. Metabolic/Cellular: 2 Hz maintained normal Ca2 + influx and mitochondrial membrane potential (*ΔΨ*m), while 4 Hz caused significant, potentially pathological decreases in both. Both frequencies increased cytosolic ATP, but levels were significantly higher with 2 Hz. Sensitization: Neither frequency elevated pERK, confirming neither induces pathological neuronal activation/sensitization in healthy cells.
Laksono et al. (20)	*In vitro*; F11 cell line differentiated into DRG neurons. Activated via NMDA (80 µM).	500 kHz. Energy-matched comparison: 2 Hz: 20 ms pulse. 4 Hz: 10 ms pulse.	Single application. 360 s.	Sensitization (pERK): 2 Hz significantly decreased pERK, normalizing pathological neuronal activity. 4 Hz showed no significant reduction in pERK. Calcium Dynamics: Both frequencies initially increased cytosolic calcium above baseline, but to a lesser extent than pure NMDA activation. 2 Hz maintained stable levels after the initial peak, whereas 4 Hz induced a continuous, gradual decline. Mitochondrial (MMP): Both 2 Hz and 4 Hz successfully and significantly reduced pathologically elevated mitochondrial membrane potential (MMP) compared to the untreated activated model.
Li et al. (21)	*In vivo* (rat); injured sciatic nerve (Chronic Constriction Injury model).	500 kHz, 45 V, 2 Hz, 20 ms pulse width. Target temp 42 °C.	Single application. 120 s.	Morphological/Cellular: PRF-treated nerves exhibited significantly less demyelination and fibrosis compared to sham controls, along with notable nerve fiber regrowth. PRF induced extensive swelling and mitochondrial hyperplasia, which the authors suggest is a compensatory metabolic response to aid in nerve regeneration. It also promoted macrophage infiltration to clear necrotic debris. Functional: PRF treatment significantly relieved both mechanical and thermal hyperalgesia 10–14 days post-treatment.
Nishioka et al. (14)	*In vitro*; human monocytic cells (THP-1). Used as a viable floating proxy possessing properties that mimic nerve cells.	480 kHz, 2 Hz, <20 ms bursts, >45 V. Temp kept <43 °C. (CRF comparison: 70 °C).	Single application. PRF: 15 min. CRF: 3 min.	Mitochondrial Homeostasis: PRF safely maintained mitochondrial membrane potential (MMP). In contrast, CRF significantly decreased MMP within 3 min. Cell Viability: PRF did not provoke apoptosis or cell death. CRF induced apoptosis within 10 min of application.
Tun et al. (15)	*In vivo* (rat); healthy brain tissue (parietal cortex) compared to sham.	PRF: 500 kHz, 2 Hz, 20 ms pulse. Target temp 42 °C. CRF: 70 °C.	Single application. PRF: 120 s. CRF: 60 s.	Light Microscopy: CRF caused central necrosis with peripheral inflammatory reaction, with the effected ratio of neurons of 14.6%. PRF caused no central necrosis and effected ratio of neurons was 5.5%. Electron Microscopy (Ultrastructural): CRF caused 83.6% of mitochondria to become edematous. PRF caused 52.7% of mitochondria to become edematous.

PRF, pulsed radiofrequency; RF, radiofrequency; CRF, constant radiofrequency; DRG, dorsal root ganglion.

### 3.1 Ultrastructure comparisons: PRF vs. CRF

Four studies specifically contrasted the mitochondrial effects of PRF against thermal CRF ablation. There was consensus across models that CRF induces severe mitochondrial pathology, described as “swollen,” “bloated,” or “edematous” mitochondria, often accompanied by cristae disruption and pore formation (13, 15). For example, Tun et al. reported that 83.6% of mitochondria in CRF-treated (70 °C for 60 s) rat brain tissue were edematous, compared to only 52.7% in the PRF group (15). Similarly, Choi et al. demonstrated that continuous RF (82 °C for 60 s) applied to the healthy rat sciatic nerve resulted in irreversible degeneration, myelinated axon collapse, absent cytoskeleton, and severe mitochondrial destruction, whereas PRF (42 °C for 120 s) induced only mild axonal damage and slight mitochondrial swelling (13). Erdine et al. further supported this in a rabbit model, noting that while light microscopy showed no pathological differences between groups, electron microscopy revealed severe mitochondrial degeneration, giant fused vacuoles, and loss of nuclear membrane integrity following CRF (67 °C), whereas PRF (<43 °C) largely preserved normal dorsal root ganglion morphology (16). This preservation extends to the immune compartment; Nishioka et al. demonstrated *in vitro* that while continuous RF (70 °C for 3 min) caused a significant decrease in mitochondrial membrane potential (MMP) and induced apoptosis within 10 min in human monocytic (THP-1) cells, PRF safely maintained their MMP and cell viability (14).

### 3.2 Impact of stimulation parameters and dosage

A mapping of PRF parameters across the included studies reveals a standardized “safety window” for mitochondrial preservation. The majority of effective nerve regeneration studies utilized the standard clinical PRF protocol: 2 Hz frequency, 20 ms pulse width, and a 42 °C temperature limit. Interestingly, while the duration of exposure per session was often standardized to 120 s in animal models, cell culture models often required extended exposures ranging from 360 s to 15 min (14, 18). Furthermore, the frequency of administration appears to influence the mitochondrial phenotype. Li et al. observed that a single 120-second session was sufficient to trigger mitochondrial hyperplasia (biogenesis) as an acute regenerative response (21). In contrast, Bayir et al. utilized a daily 120-second protocol for 30 days, resulting in the sustained preservation of mitochondrial ultrastructure (12). However, this study also reported no significant improvement in functional motor recovery (Sciatic Functional Index) over 4 weeks compared to controls, highlighting that histological preservation does not immediately guarantee functional clinical recovery. This suggests that while a single dose may initiate the regenerative cascade, repeated dosing may support the maintenance of mitochondrial integrity during the lengthy process of nerve repair. Regarding voltage and frequency, higher electrical field strengths were associated with differential mitochondrial responses. Nishioka et al. reported that while continuous RF caused apoptosis, PRF at standard voltages (>45 V) maintained mitochondrial membrane potential in monocytic cells (14). However, Erdine et al. also cautioned that unmyelinated C-fibers may be vulnerable to ultrastructural damage (17). Notably, this specific study employed an atypical 1 ms pulse width rather than the standard 20 ms, suggesting a lower threshold for mitochondrial stress in small-diameter sensory fibers when exposed to altered pulse parameters compared to large, myelinated motor fibers.

### 3.3 Modulation of mitochondrial bioenergetics

Several studies investigated the functional bioenergetics of mitochondria following PRF, focusing on membrane potential (*ΔΨm*), ATP production, and calcium mobility. In a series of experiments on F11 cell lines differentiated into Dorsal Root Ganglion (DRG) neurons, Laksono et al. demonstrated that PRF effects are state-dependent (18–20). In “sensitized” (NMDA-activated) DRG models (mimicking neuropathic pain), PRF significantly decreased pathologically elevated mitochondrial membrane potential and calcium influx while increasing cytosolic ATP, effectively reversing the bioenergetic profile associated with sensitization. Interestingly, these effects varied by frequency. Laksono et al. found that while 4 Hz PRF decreased calcium influx and membrane potential in healthy neurons, 2 Hz PRF did not, though both frequencies elevated cytosolic ATP (19). This suggests that PRF may act as a “metabolic modulator,” boosting energy availability without driving excitotoxic calcium influx. Furthermore, Laksono et al. demonstrated that in activated DRG neurons, 2 Hz PRF significantly decreased pERK levels (normalizing pathological neuronal activity), whereas 4 Hz completely failed to reduce pERK (20). This suggests that PRF may act as a “metabolic modulator,” boosting energy availability without driving excitotoxic calcium influx, provided the correct frequency (e.g., 2 Hz) is utilized.

### 3.4 Mitochondrial hyperplasia and regeneration

Beyond bioenergetics, PRF appears to stimulate mitochondrial biogenesis as a mechanism for nerve repair. Li et al. reported that in a rat model of chronic constriction injury, PRF-treated nerves exhibited significantly less severe demyelination, fibrosis, and Schwann cell proliferation compared to the untreated sham group (21). Furthermore, PRF promoted macrophage infiltration to clear necrotic debris, resulting in significant relief of mechanical and thermal hyperalgesia 10-14 days post-treatment. Crucially, PRF-treated nerves displayed “mitochondrial hyperplasia” (an increase in number). While some mitochondrial swelling was also observed in these nerves, this phenotype stood in sharp contrast to the untreated group, where mitochondria were “greatly reduced” or destroyed. The authors postulated that this hyperplasia serves as a compensatory metabolic response to meet the increased metabolic demand required for regeneration. This aligns with findings by Bayir et al., where PRF treatment similarly preserved Schwann cell organelles and supported the reorganization of neurofilaments, linking mitochondrial integrity to structural repair (12). It also aligns with observations by Choi et al., who noted that PRF only transiently upregulated pro-inflammatory markers (e.g., GFAP, TNF-α, IL-6), which recovered to normal by day 30, supporting a temporary, restorative inflammatory response rather than persistent pathological inflammation (13).

## 4 Discussion

This scoping review synthesizes evidence from ten studies to propose that the therapeutic effects of Pulsed Radiofrequency (PRF) stimulation may be mediated, at least in part, by the potential modulation of mitochondrial dynamics. While PRF is clinically established as a non-destructive alternative to continuous RF ablation, the cellular basis for its analgesic and regenerative effects has remained elusive. Our synthesis identifies three potential mitochondrial mechanisms driven by PRF: i) the preservation of mitochondrial ultrastructure compared to ablative RF; ii) the acute modulation of bioenergetics (membrane potential and Ca^2+^ flux) to dampen excitability; and iii) the induction of mitochondrial biogenesis to support long-term nerve regeneration.

### 4.1 Mitochondria as a potential pivot point for analgesia

The central finding from recent *in vitro* work (18–20) is that PRF directly alters mitochondrial membrane potential (*ΔΨm*) and calcium handling in dorsal root ganglion (DRG) neurons. This provides a biophysical explanation for the “anti-inflammatory” effects often attributed to PRF. In neuropathic pain states, mitochondrial dysfunction leads to excessive Reactive Oxygen Species (ROS) production and impaired calcium buffering, which drives central sensitization and hyperexcitability (22, 23). This dysfunction is intimately linked to the interruption of normal mitochondrial dynamics. Specifically, elevated levels of mitochondrial fission, the division of mitochondria often triggered by cellular stress and increased energy demand, are observed during the acute phase of neuropathic pain and contribute heavily to this pathological ROS overproduction (24). By depolarizing the mitochondrial membrane and modulating calcium influx, PRF appears to interrupt this “energy crisis.” This mechanism aligns with findings by Yuba et al. who demonstrated that PRF reduces the axonal transport of pro-nociceptive neuropeptides (CGRP, Substance P) (25). We propose that this acute analgesic effect is metabolically driven: PRF restores mitochondrial calcium homeostasis, thereby reducing the ATP-dependent axonal transport of pain transmitters and dampening the ROS signaling that sustains chronic inflammation.

### 4.2 Ultrastructural preservation, intracellular transfer, and regeneration

A critical distinction emerged regarding mitochondrial morphology when contrasting PRF with continuous RF ablation. Studies applying RF to healthy, naive tissues, such as Choi et al. and Tun et al. consistently demonstrate that continuous RF acts as a neuro-destructive lesion, inducing severe Wallerian degeneration, coagulative necrosis, and complete mitochondrial vacuolization (13, 15). By contrast, the ultrastructural profile observed with PRF suggests a fundamentally different, potentially restorative effect. For instance, Li et al. reported distinct “mitochondrial hyperplasia” following PRF treatment in a nerve injury model (21). While this hyperplasia was accompanied by some swelling, the overall phenotype differed significantly from the untreated injury state, which was characterized by mitochondrial loss and extensive degeneration. The authors attributed this phenomenon to a compensatory mechanism required to meet the metabolic demands of nerve regeneration. However, this must be interpreted with caution. Because these studies relied primarily on ultrastructural imaging (such as transmission electron microscopy), which captures physical characteristics but cannot definitively identify biochemical intent, it is challenging to definitively state whether the observed swelling and hyperplasia represent healthy compensatory biogenesis or precede cellular stress cascades like mitophagy or ferroptosis (26, 27). Definitively distinguishing these pathways requires future *in vivo* PRF studies utilizing targeted molecular profiling.

Peripheral nerve regeneration is energetically expensive, requiring vast amounts of ATP for remyelination and cytoskeletal reorganization (28). Crucially, understanding PRF-driven regeneration requires viewing the nerve as a cohesive metabolic unit, where sensory neurons do not meet this demand in isolation. Schwann cells are metabolically coupled to axons, supplying lactate for mitochondrial ATP production (29), while recent breakthrough evidence demonstrates that satellite glial cells (SGCs) and macrophages actively transfer healthy mitochondria to neurons via tunneling nanotubes to rescue them from neuropathy (30, 31). The observed increase in mitochondrial density following PRF treatment in Li et al. likely represents the preservation or enhancement of this critical neuro-glial crosstalk (21). This hypothesis is supported by the stark contrast in outcomes: while continuous RF leads to necrosis and mitochondrial vacuolization in neural tissues and apoptosis in monocytic immune cells, PRF maintains the integrity of Schwann cells and neurofilaments and preserves the mitochondrial membrane potential of monocytes. By protecting these supportive glial and immune networks rather than ablating them, PRF likely ensures the continuous supply of metabolites and intact organelles necessary to fuel long-term axonal repair, fostering an environment conducive to regeneration.

### 4.3 Connecting mitochondrial dynamics to clinical onset

The dual-phase mitochondrial mechanism proposed here offers a biological explanation for the variable onset of analgesia observed in clinical practice. The “acute phase”, mediated by immediate depolarization of the mitochondrial membrane and modulation of calcium influx, aligns with reports of immediate pain relief following PRF, likely due to a rapid interruption of nociceptive transmission and axonal transport (18, 25). However, the “regenerative phase” relies on mitochondrial biogenesis, a process that is biologically slow. Li et al. noted that in a rat model of chronic constriction injury, significant behavioral analgesia and structural repair were not observed until 10–14 days post-treatment (21). This 10–14-day lag time closely aligns with the established timescale for Wallerian degeneration and subsequent myelin debris clearance in rodent models (32, 33). Rather than dictating this timeline, the delayed onset of analgesia could reflect the immense metabolic toll of the broader nerve regeneration process. The observed mitochondrial hyperplasia likely serves as the critical bioenergetic support system required to fuel the ATP-intensive processes of clearing Wallerian debris, cytoskeletal reorganization, and axonal sprouting over this two-week period. Clinically, this suggests that PRF outcomes should be evaluated over a distinct timeline: immediate effects may reflect bioenergetic modulation, while long-term recovery (weeks) reflects mitochondrial-driven structural regeneration. This distinction is critical for setting patient expectations and designing follow-up protocols.

## 5 Conclusion

This review provides the first systematic map linking PRF stimulation to mitochondrial function. The evidence challenges the view of PRF as merely a “gentler” version of ablation, but also cautions against over-promising its efficacy, as histological improvements do not always guarantee immediate functional recovery. To fully harness these mechanisms and translate them into clinical practice, future research must transition from morphological assessments toward targeted *in vivo* tracking of specific mitochondrial biomarkers. By bridging this translational gap, the field can shift toward PRF parameters optimized specifically to maximize mitochondrial efficiency and neural regeneration, rather than relying on protocols designed solely to avoid thermal damage.
